# Accurate, explicit formulae for higher harmonic force spectroscopy by frequency modulation-AFM

**DOI:** 10.3762/bjnano.6.14

**Published:** 2015-01-13

**Authors:** Kfir Kuchuk, Uri Sivan

**Affiliations:** 1 Department of Physics and The Russell Berrie Nanotechnology Institute, Technion – Israel Institute of Technology, Haifa 32000, Israel

**Keywords:** atomic force spectroscopy, higher harmonic FM-AFM

## Abstract

The nonlinear interaction between an AFM tip and a sample gives rise to oscillations of the cantilever at integral multiples (harmonics) of the fundamental resonance frequency. The higher order harmonics have long been recognized to hold invaluable information on short range interactions but their utilization has thus far been relatively limited due to theoretical and experimental complexities. In particular, existing approximations of the interaction force in terms of higher harmonic amplitudes generally require simultaneous measurements of multiple harmonics to achieve satisfactory accuracy. In the present letter we address the mathematical challenge and derive accurate, explicit formulae for both conservative and dissipative forces in terms of an arbitrary single harmonic. Additionally, we show that in frequency modulation-AFM (FM-AFM) each harmonic carries complete information on the force, obviating the need for multi-harmonic analysis. Finally, we show that higher harmonics may indeed be used to reconstruct short range forces more accurately than the fundamental harmonic when the oscillation amplitude is small compared with the interaction range.

## Introduction

AFM measurements are presently utilized to generate atomic resolution [1–2], 3D force maps that carry unprecedented information on the interfacial properties of soft matter [3], water structure [1–2] and ion ordering [4]. The generation of such force maps relies invariably on AC detection methods, most commonly at frequencies in the vicinity of the cantilever’s fundamental resonance frequency. In frequency modulation-AFM (FM-AFM), the force is usually reconstructed from the resonance frequency shift, which in the small amplitude regime is proportional to the derivative of the force with respect to tip–surface distance. Similarly, it has been recognized that higher harmonics generated by the nonlinear tip–surface interaction (to be distinguished from higher flexural modes of the cantilever) are related to higher derivatives of the force, and thus carry additional information on the interaction [5–11]. Broad implementation of force spectroscopy by analysing higher harmonics has been nevertheless impeded by the lack of a closed-form expression for the force in terms of measured quantities, namely, the lack of a higher harmonics analogue of the Sader–Jarvis formula for the fundamental harmonic [12–13]. In the present letter we fill this gap by providing such formulae for both conservative and dissipative forces.

In FM-AFM, a cantilever is oscillated at its resonance frequency using an external driving force and a feedback loop. The motion of the cantilever is often modelled as a driven damped harmonic oscillator with an additional force, *F*_ts_, stemming from tip–surface interaction

[1]
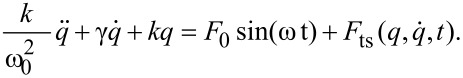


Here, *k* is the effective cantilever spring constant, ω_0_ is the fundamental resonance frequency in the absence of tip–surface interaction, *q*(*t*) is the tip position, γ is the damping coefficient, and *F*_0_ and ω are the amplitude and frequency of the driving force, respectively. As the cantilever is brought close to a surface, the tip–surface interaction forces shift the resonance frequency. The relation between the frequency shift and *F*_ts_, in the case where the force depends only on tip position, was first derived by Giessibl as [14]

[2]
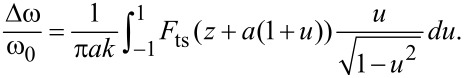


Here, Δω is the frequency shift, *a* is the oscillation amplitude and *z* is the distance of closest approach to the surface in the oscillation cycle. Various techniques have been proposed to invert the convolution in Equation 2 and extract the interaction force from the measured shift in frequency. At first, these were either numerical solutions or analytic approximations of large or small amplitudes [15], but later Sader and Jarvis derived an interpolation formula which is valid for all amplitudes [12–13] (the Sader–Jarvis formula). Its application has also been extended to AM-AFM [16].

Expressions similar to Equation 2, relating the Fourier components of higher harmonics to a convolution over *F*_ts_, have been derived [15,17], but existing methods to recover *F*_ts_ from higher harmonics rely on spectral analysis of the AFM signal [18–19], and require the measurement of a significant number of harmonics to obtain reasonable accuracy [5,17]. Although measurement of all spectral components would theoretically enable fast reconstruction of the force while scanning, the simultaneous acquisition of many harmonics is demanding and requires multiple lock-in amplifiers. For the generation of 3D force maps, the multi-harmonic reconstruction is further complicated since multiple scans must be performed at different heights. Additionally, higher harmonic amplitudes decrease rapidly with harmonic number, limiting the number of measurable harmonics and, hence, the accuracy of force reconstruction. Some methods to amplify the signals of higher harmonics have been exercised [6,20], but these do not completely alleviate the problem. Here, we show that a full force curve can be extracted from the amplitude of any single higher harmonic. We provide simple, explicit expressions for the interaction force in terms of higher harmonic amplitudes, allowing the benefits of high-harmonics force spectroscopy with no need for multiple-harmonics measurements and analysis.

There are several advantages to be gained by expressing the force as a function of higher harmonic amplitudes. First, the existence of these amplitudes depends entirely on the presence of nonlinear interaction forces. Higher harmonic amplitudes may therefore be measured with greater precision compared with fundamental harmonic measurements. While the former are measured with reference to zero, the latter are obtained by offsetting large, inherently noisy signals, such as the driving frequency in FM-AFM or oscillation amplitude in AM-AFM. Second, there is evidence [5–11] that higher harmonics are more sensitive to short-range forces than the fundamental harmonic. This becomes evident when the cantilever oscillation amplitude is small compared with the interaction length. As we show, the frequency shift in this case is related to the first derivative of the force, while higher harmonics are related to higher derivatives. The *n*th harmonic therefore probes directly the *n*th derivative of the force, enhancing the sensitivity to short range forces. The main difficulty in measuring higher harmonics is their small magnitude due to the weak response of the cantilever to frequencies far from its resonance. At these frequencies, the noise in well-designed AFMs is dominated by the shot noise of the photodiode in the optical detection system. The SNR of higher harmonic amplitudes is therefore expected to deteriorate with harmonic order, but in many cases a significant number of higher harmonics can still be measured [10,21]. Unlike previous higher harmonic reconstruction methods, the disclosed scheme can be applied also to cases where only a few harmonics are measurable.

## Results and Discussion

Consider Equation 1 with the interaction force expressed by its Fourier components:

[3]
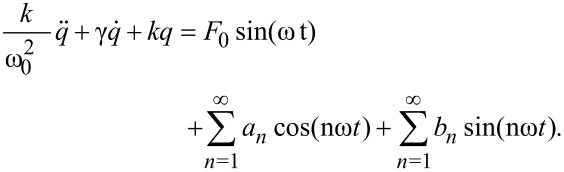


The cantilever motion is assumed to be nearly that of a free harmonic oscillator with small harmonic contributions, μ*_n_* and ν*_n_*, generated by the nonlinear tip–surface interaction

[4]
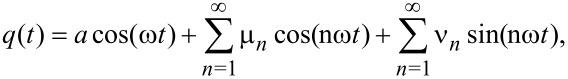


with μ*_n_*/*a* and ν*_n_*/*a* << 1 for all *n*.

We begin by analysing the even, conservative part of the force [13], which depends only on tip–sample separation. Substituting Equation 4 into Equation 3 and using orthogonality, one arrives at the relation

[5]
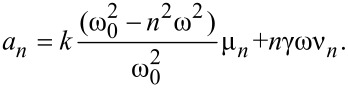


Using the definition of *a_n_* and changing variables one finds

[6]
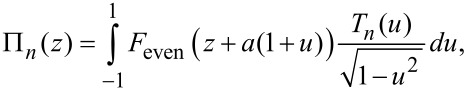


where

[7]
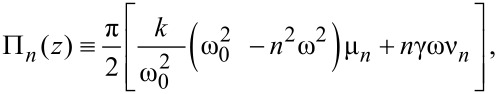


and *T_n_*(*u*) = cos(*n*cos^-1^(*u*)) is the *n*th order Chebyshev polynomial of the first kind. As expected, by setting γ = 0 in (7), we recover the result obtained by Dürig [17].

To invert the integral in Equation 6 and express the force in terms of the measured amplitudes μ*_n_* and ν*_n_*, we generalize the derivation of the Sader–Jarvis formula [12] to an arbitrary harmonic, *n*. First, we express *F*_even_ in terms of its inverse Laplace transform, 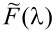:

[8]
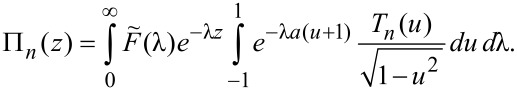


Using the integral representation [22] of *I_n_*, the *n*th order, modified Bessel function of the first kind, along with the Rodrigues’ representation [22] of *T_n_*,

[9]
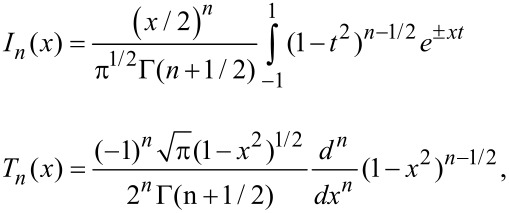


the integral over *u* can be evaluated

[10]
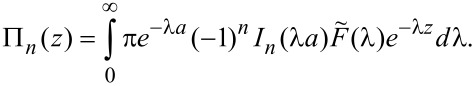


Comparison between Π*_n_* and *F*_even_ in Laplace space shows that

[11]
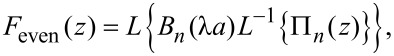


where *B_n_*(*x*) = (−1)*^n^*e*^x^*/*πI_n_*(*x*). Making use of the asymptotic forms [22] of *I_n_*, an approximation to *B_n_*(*x*) is constructed

[12]
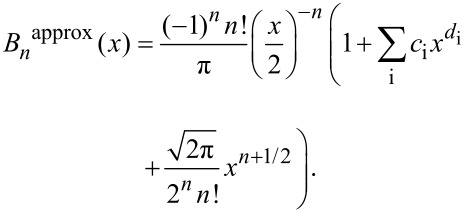


For 0 < *d*_i_ < *n*+1/2, Equation 12 has the correct asymptotic behaviour for very small and very large *x*. An arbitrary number of terms of the form 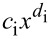, for some set of coefficients *c*_i_, can be fitted to improve the accuracy of Equation 12 as needed. This is in fact what the Sader–Jarvis formula does for the fundamental harmonic – it interpolates between the regimes of large and small amplitudes, where analytic solutions exist, by fitting terms in the intermediate regime.

Substituting Equation 12 into Equation 11 and using the following results of fractional calculus [12],

[13]
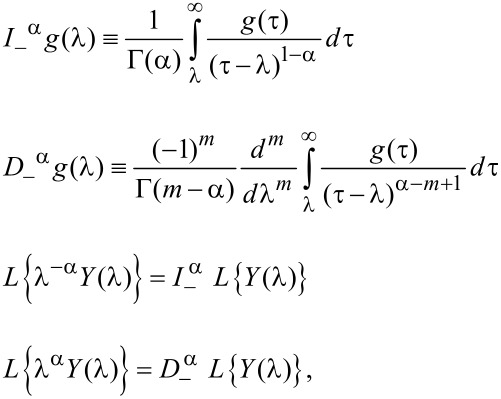


where α > 0 and *m* = [α] + 1, the force is expressed explicitly in terms of the interpolation parameters *c*_i_, *d*_i_. In particular, if *d*_i_ are chosen to be integers, the force is given by

[14]
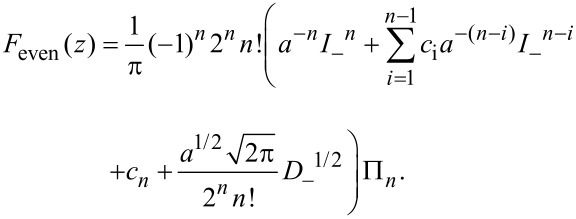


The force may thus be derived in terms of any harmonic, provided that the interpolation coefficients *c*_i_ approximate *B_n_* sufficiently well. Explicit formulae for the force in terms of harmonics 2–6 of the fundamental frequency are given in Table 1, where the interpolation coefficients were calculated such that 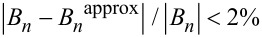 for all positive *x*. The force formulae in terms of other higher harmonics may be derived in a similar way. In the special case *n* = 2 (Equation 15),

[15]
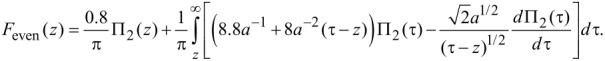


A similar procedure can be applied to recover the odd, dissipative, part of the force from higher harmonics, but a subtlety must first be addressed. The derivation of *F*_even_ relies on its sole dependence upon tip–sample separation in Equation 6. This is not the case for *F*_odd_, which is out of phase with *q*(*t*). This issue is resolved by noting that many dissipative forces have the form [13]

[16]
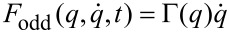


with Γ, the generalised damping coefficient, depending only upon tip–sample separation. It then follows from Equation 3 that

[17]
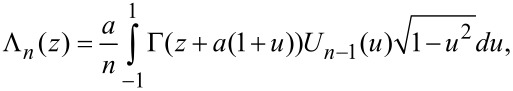


where 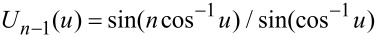 is the *n*th order Chebyshev polynomial of the second kind and

[18]
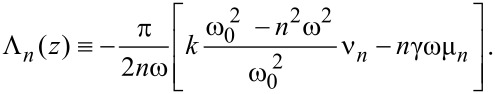


Integrating by parts and using the identity 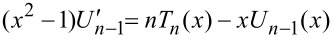, Equation 17 assumes the form

[19]
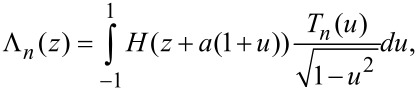


where 
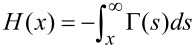
. Comparing Equation 19 with Equation 6, we see that these expressions are identical and therefore have the same solutions. We may then refer to Table 1 for these solutions. For example, using Equation 15, the formula for the generalized damping coefficient for *n* = 2 is readily derived as (Equation 20):

[20]
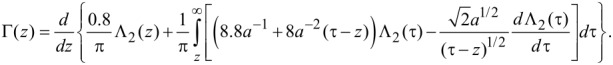


Expressions in terms of higher harmonics may be similarly derived.

**Table 1:** Formulae for the force in terms of harmonics 1–6. The Sader–Jarvis formula for *n* = 1 is given here for completeness. An implementation of these formulae is available in the supplementary Mathematica file.

*n*	*F*_even_ in terms of the *n*th harmonic

1	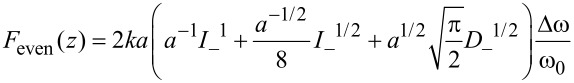
2	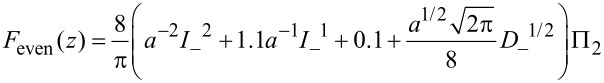
3	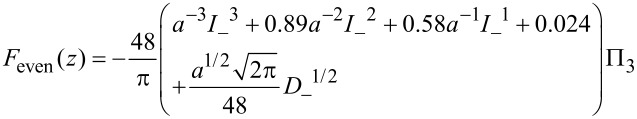
4	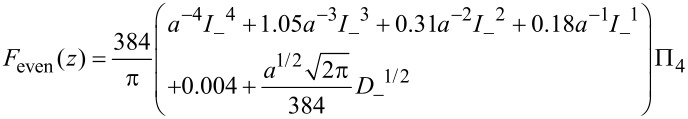
5	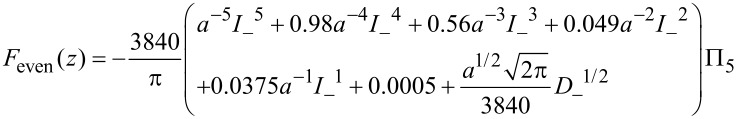
6	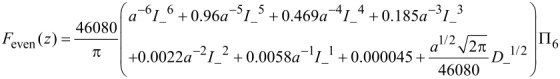

We have shown that by measuring any pair of higher harmonic amplitudes, μ*_n_* and ν*_n_*, the full force profile can be recovered. However, the same information can be derived by analysing the first harmonic frequency shift. This begs the question, what new information have we gained in the process? Several experiments [5,7–8] show that for small oscillation amplitudes, higher harmonics enhance the sensitivity to short range interactions compared with first harmonic FM-AFM. This sensitivity has been reasoned by an expression derived by Giessibl [6], which relates the *n*th harmonic to a convolution over the *n*th derivative of the interaction force. In the small oscillation amplitude limit, the two are proportional. Using Equation 14, we reaffirm this relation. For small amplitudes, Equation 14 is dominated by the term proportional to *a*^−^*^n^*, and the even force term can be approximated by

[21]
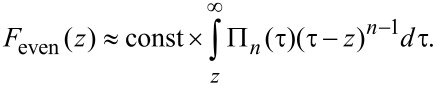


Integrating Equation 21 by parts and then differentiating *n* times with respect to *z* one finds

[22]
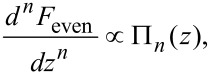


which is similar to the small amplitude approximation derived from Equation 2

[23]
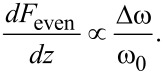


As expected, higher harmonics are proportional to higher derivatives, while the frequency shift used in first harmonic force spectroscopy is proportional to the first derivative. This suggests that reconstruction of the force using higher harmonics is more sensitive to short range forces compared with reconstruction using the Sader–Jarvis formula, as long as the oscillation amplitude is small compared with the characteristic interaction length.

To test the accuracy of our force inversion formulae, we insert a known conservative force into Equation 6, a generalized damping coefficient into Equation 17, and then recover them with second harmonic analysis, namely with Equation 15 and Equation 20. For the conservative interaction, we employ a Lennard–Jones force law

[24]
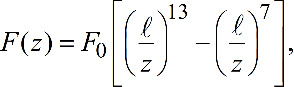


Where *F*_0_ is constant and ℓ is the interaction length scale. For the dissipative interaction, we use a viscoelastic type of force [23], characterized by the generalized damping coefficient

**Figure 1:**
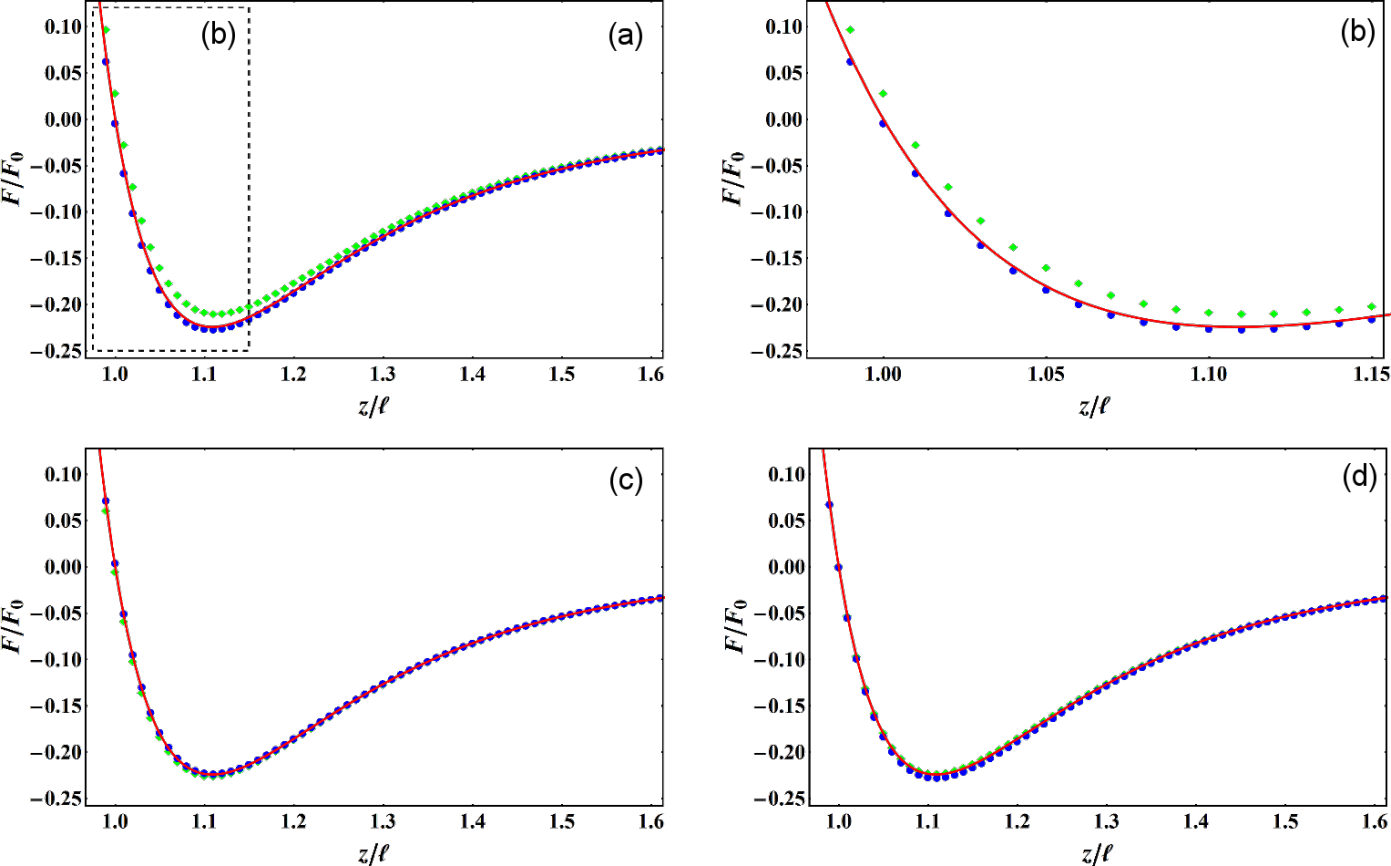
Lennard–Jones interaction (solid red line) and reconstructed forces. Blue circles depict reconstruction using the second harmonic. Green diamonds depict reconstruction using the Sader–Jarvis formula for the fundamental harmonic. Amplitudes of oscillation used are 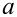/ℓ = 0.1 (a), 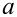/ℓ = 1 (c), 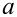/ℓ = 10 (d). (b) depicts magnification of the dashed frame marked in (a).

**Figure 2:**
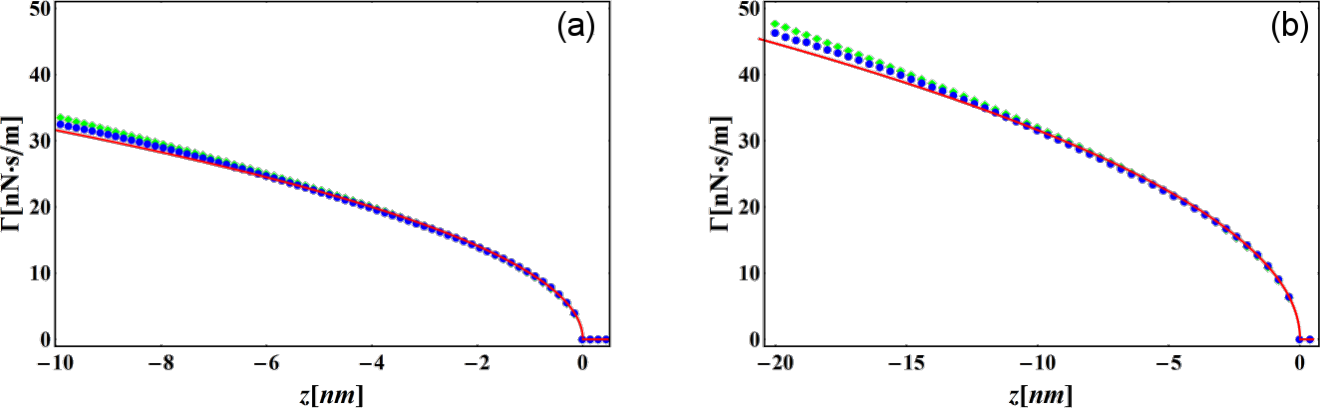
Generalized damping coefficient of a viscous interaction (solid red line) and its reconstructions. Blue circles depict reconstruction using the second harmonic. Green diamonds depict reconstruction using the Sader–Jarvis formula for the fundamental harmonic. The tip radius and viscosity are *R* = 10 nm and η = √10 Pa·s. The amplitudes of oscillation are 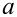 = 10 nm (a) and 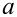 = 20 nm (b).

[25]
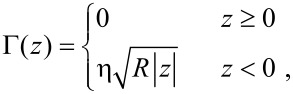


where η is the viscosity, *R* is the tip radius, and the sample surface is assumed to be at *z* = 0. The results displayed in Figure 1 and Figure 2 demonstrate the accuracy of our formulae. Figure 1a,b confirms, in the small amplitude regime, the increased sensitivity to short range interaction of force reconstruction using higher harmonics compared with the Sader–Jarvis formula. As the oscillation amplitude grows smaller compared with the range of the Lennard–Jones potential, the Sader–Jarvis formula grows inaccurate, while reconstruction using the second harmonic maintains its accuracy. When the amplitude is increased (Figure 1c), the accuracy of the Sader–Jarvis formula improves and for large amplitudes (Figure 1d) both methods yield satisfactory results. Figure 2 depicts the reconstruction of the generalized damping coefficients. Both the Sader–Jarvis formula for dissipative forces and the second harmonic reconstruction lose accuracy as the indentation into the simulated surface increases to the order of the oscillation amplitude, but second harmonic reconstruction remains the more accurate of the two.

## Conclusion

We have derived a general procedure yielding both conservative and dissipative forces in terms of cantilever oscillations at an arbitrary harmonic, and provided explicit formulae for harmonics 2–6. This procedure reconstructs the full interaction force curve from any single harmonic, obviating existing reconstruction methods based on simultaneous measurement of multiple higher harmonics. In addition, it was shown that in the small amplitude regime, short range forces are reconstructed more accurately by higher harmonic analysis compared with the fundamental harmonic one.

## Supporting Information

A supplementary Mathematica notebook file containing an implementation of the formulae of Table 1 for reconstruction of simulated conservative forces can be found in the ZIP file of the Supporting Information.

File 1 Force reconstruction.
